# Extracellular gp96 is a crucial mediator for driving immune hyperactivation and liver damage

**DOI:** 10.1038/s41598-020-69517-7

**Published:** 2020-07-28

**Authors:** Zeliang Guan, Yun Ding, Yongai Liu, Yu Zhang, Jingmin Zhao, Changfei Li, Zihai Li, Songdong Meng

**Affiliations:** 1 Key Laboratory of Pathogenic Microbiology and Immunology, Institute of Microbiology, Center for Biosafety Mega-Science, Chinese Academy of Sciences (CAS), Beijing, China; 2 University of Chinese Academy of Sciences, Beijing, China; 3 Heilongjiang Bayi Agricultural University, Heilongjiang, China; 4 Department of Pathology and Hepatology, The 5th Medical Centre, Chinese PLA General Hospital, Beijing, China; 5 Pelotonia Institute for Immuno-Oncology, The Ohio State University, Columbus, OH USA

**Keywords:** Cell death and immune response, Inflammation, Liver diseases, Hepatitis

## Abstract

Liver failure leads to the massive necrosis of hepatocytes, releasing large amounts of intracellular components including damage-associated molecular patterns (DAMPs). We found that extracellular gp96 levels in serum were elevated in patients with chronic hepatitis B infection (CHB) and acute-on-chronic liver failure (ACLF). Meanwhile, the gp96 level positively correlated with hepatic necroinflammation. We employed two mouse liver damage and liver failure models induced by lipopolysaccharide (LPS) plus d-galactosamine (d-Galn), and concanavalin A (ConA) to identify the function of extracellular gp96. As a result, the inhibition of extracellular gp96 by a specific peptide efficiently mitigated both LPS/d-Galn- and ConA-induced liver injury and immune hyperactivation, whereas exogenous gp96 aggravated the symptoms of hepatic injury in mice but not in Kupffer cells-ablated mice. The exposure of Kupffer cells to gp96 induced the secretion of pro-inflammatory cytokines. Collectively, our data demonstrate that gp96 released from necrotic hepatocytes aggravates immune hyperactivation and promotes liver damage and possibly the development of liver failure mainly by activating Kupffer cells.

## Introduction

Liver failure is a syndrome characterized by the loss of the organ’s normal synthetic and metabolic functions, accompanied by many other complications. Liver failure is one of the most severe liver diseases, with no effective prevention or treatment strategies^1^. During the development of liver failure, massive hepatocyte necrosis is the main event, which may be induced by direct injury to hepatocytes resulting from the uptake of chemicals or drugs, or it can be induced by immune-mediated injury during virus infections. Acute-on-chronic liver failure (ACLF) represents a typical clinical syndrome in patients with chronic liver disease and cirrhosis, and chronic hepatitis B (CHB) is one of the major causes of ACLF^2–4^. Hyperactivation of the immune system is considered to greatly contribute to the pathogenesis of liver failure. Hyperactivation of immune cells, including Kupffer cells, T cells, and natural killer (NK) cells, as well as the infiltration of large amounts of neutrophils in the liver, can aggravate the apoptosis or necrosis of hepatocytes. Inflammatory cytokines secreted by these immune cells, including TNF-α, IL-6, IFN-γ, and IL-1β, also contribute to the immune-mediated injury, in which TNF-α is considered to play a major role in cytokine-induced liver damage^5–7^. TNF-α both induces the apoptosis and necrosis of hepatocytes and stimulates the production of other inflammatory cytokines^8^.

In addition, intracellular components released by necrotic cells initiate hepatic innate immune responses and lead to sterile inflammation through damage-associated molecular patterns (DAMPs), which include nucleic acids, adenosine triphosphate (ATP), proteins, and mitochondrial compounds^9^. For instance, high mobility group box 1 (HMGB1), a highly conserved nuclear protein expressed in almost all eukaryotic cells, is passively released as a DAMP in case of necrotic cell death and interacts with TLR4 to initiate the early stage of nonalcoholic fatty liver disease(NAFLD)^10^. Cyclophilin A (CypA) is an abundant cytosolic protein that mediates protein folding. After cell death, the released CypA can aggravate acetaminophen-induced liver injury^11^. These findings have led to a thorough understanding of the pathophysiology of liver failure, which has contributed to the identification of new rational therapeutic targets.

As a member of heat shock protein (HSP) 90 family, gp96 is one of the most abundant cellular proteins and is normally located in the endoplasmic reticulum (ER). Aside from being a key molecular chaperon mediating its client protein folding and assembly in the ER, it also plays an important role in regulating innate and adaptive immunity^12–17^. First, intracellular gp96 takes part in antigen presentation to major histocompatibility complex (MHC) molecules in the ER^18^. Meanwhile, extracellular gp96-peptide complexes may be taken up by antigen presenting cells (APCs), and the associated peptides can be cross-presented to MHC I molecules for CD8^+^ T cell activation^19^. Second, gp96 itself can prime and activate multiple immune cells and induce the secretion of inflammatory cytokines by interacting with its receptors CD91, toll like receptor(TLR)2/4 and promoting the downstream NF-κB pathway, or activating NLRP3 inflammasomes of antigen presenting cells (APCs)^13,20–22^. Recently, several lines of evidence indicate that aberrantly elevated levels of extracellular gp96 are associated with chronic inflammation and autoimmune diseases. Pope et al*.* discovered that high levels of gp96-IgG complexes exist in the synovial fluids of patients with rheumatoid arthritis (RA), and these gp96 molecules can activate macrophages to secrete inflammatory cytokines such as IL-6 and TNF-α^23^. Patients with type 1 diabetes^24^ or with gastrointestinal graft-versus-host disease (GVHD)^25^ exhibit high levels of circulating gp96, and gp96 levels correlate with disease severity, supporting the notion that extracellular gp96 is involved in autoimmune conditions. Additionally, Lewis et al*.* found that exogenous gp96 leads to the activation of immune cells in vivo, which in turn leads to the reduction of survival time of mice with pancreatic islet transplantation^26^. Li et al*.* also described that DCs expressing aberrant membrane gp96 by genetically engineering can release high levels of inflammatory cytokines, resulting in systemic lupus erythematosus (SLE) in mice^27^.

The highly abundant gp96 chaperon acts as a central component of the cellular protein quality control system in hepatocytes. We recently found that chronic inflammation and viral infections further induce its expression in the liver^28^. During liver failure, the normally ER-resident gp96 may be released into the extracellular microenvironment after hepatic necrosis. Given the ability of gp96 to act as a DAMP and induce aberrant immune activation, the aim of this study was to determine the potential role of extracellular gp96 in regulating immune-induced liver injury during liver failure. The results may offer new therapeutic strategies for immune hyperactivation, liver injury and liver failure.

## Results

### Serum gp96 levels are elevated in patients with CHB and ACLF

**Figure 1:**
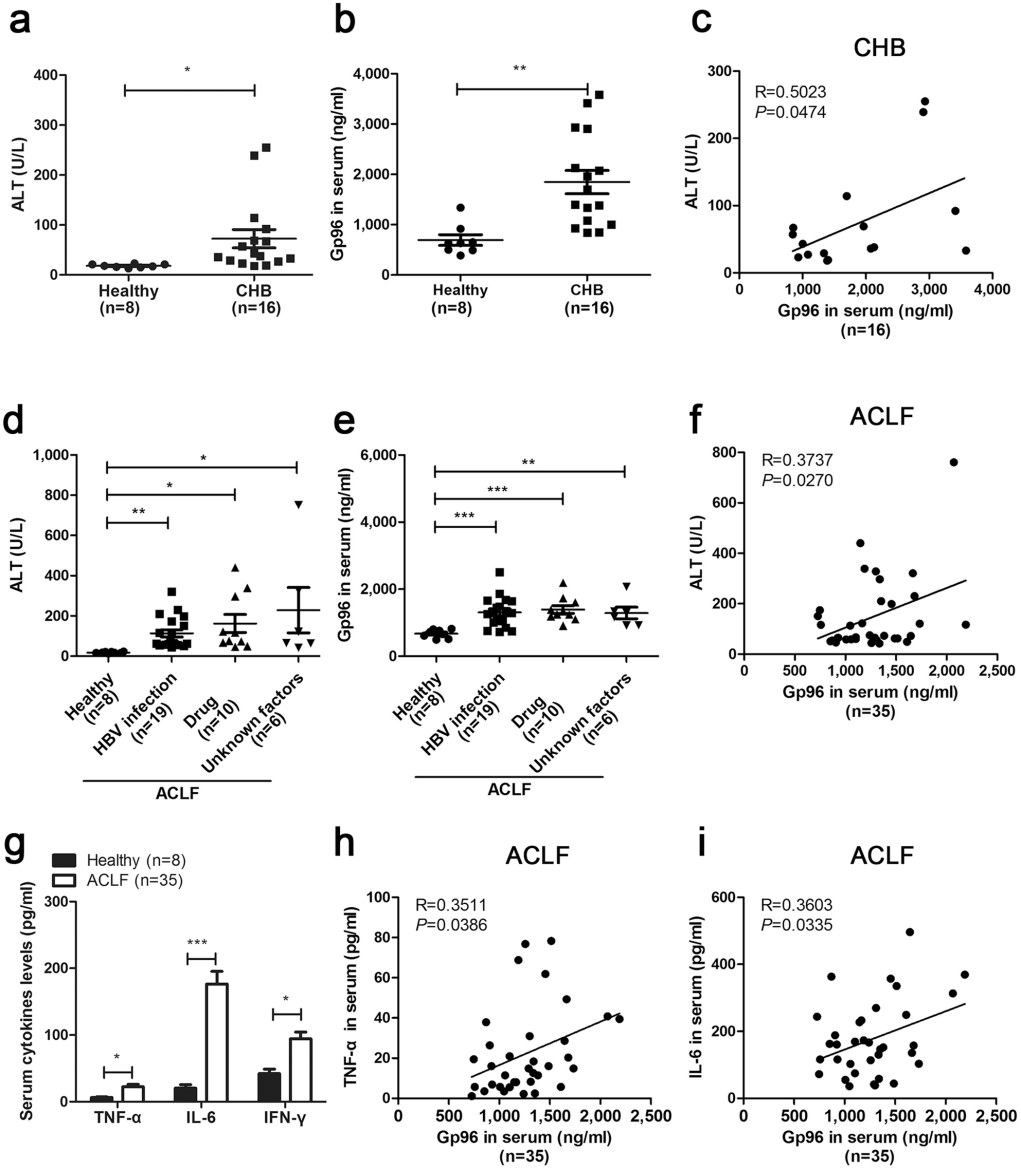
Serum gp96 levels are elevated in patients with CHB and ACLF. (**a**) Serum ALT or (**b**) gp96 levels in patients with CHB and healthy controls were measured by ELISA. (**c**) Correlation analysis between serum ALT and gp96 levels in patients with CHB. Pearson’s correlation coefficient (R) and *P*-value were analysed. (**d**) Serum ALT or (**e**) gp96 levels in patients with ACLF induced by HBV infection, drug, or unknown factors were measured by ELISA. (**f**) Correlation analysis between serum ALT and serum gp96 levels in patients with ACLF. (**g**) Serum cytokines levels of TNF-α, IL-6, and IFN-γ in patients with ACLF were measured by ELISA. Correlation analysis between serum TNF-α (**h**) or IL-6 (**i**) and serum gp96 levels in patients with ACLF. Data are presented as mean ± SEM. *p < 0.05, **p < 0.01, and ***p < 0.001 compared to the control.

As intracellular gp96 expression has been shown to be induced by HBV infection and chronic inflammation in both CHB and mouse models^28^, we first determine whether extracellular gp96 is affected by CHB or ACLF. Sixteen patients with CHB and 35 patients with ACLF (Table 1) were enrolled for the analysis of serum gp96 levels. We observed that serum alanine aminotransferase (ALT) and gp96 levels in patients with CHB (Fig. 1a,b) or HBV infection-, drug, or unknown factor-induced ACLF (Fig. 1d,e) were both higher than those in healthy controls. There was a significant correlation (p = 0.0474) between serum gp96 levels and ALT levels in CHB (Fig. 1c) and ACLF (Fig. 1f). In addition, serum aspartate aminotransferase (AST) levels in ACLF also demonstrated a correlation with serum gp96 (Supplementary Fig. S1a,b). Serum pro-inflammatory cytokine levels (including TNF-α, IL-6, and IFN-γ) were elevated compared to healthy controls (Fig. 1g). As it can been seen in Fig. 1h and i, there was also a correlation between serum gp96 and TNF-α or IL-6 levels in patients with ACLF, indicating that serum gp96 may be associated with immune hyperactivation.

**Table 1:** Clinical characteristics of patients with CHB and ACLF.

Characteristics	Value(s) for				
	Healthy	CHB	ACLF		
			HBV-associated	Drug-induced	Unknown factors-induced
No. of patients	8	16	19	10	6
Age (year; means ± SD)	32.1 ± 7.3	32.9 ± 12.5	40.3 ± 10.2	45.6 ± 14.7	37.2 ± 17.5
Gender (no. of M/F)	5/3	8/8	17/2	5/5	5/1
ALT (U/L) (means ± SD)	18 ± 3.4	70 ± 77.2	113.5 ± 79.0	162.4 ± 141.9	230.3 ± 280.5
AST (U/L) (means ± SD)	23.3 ± 7.7	–	142.2 ± 91.2	152.2 ± 85.9	101.2 ± 58.4

*CHB* chronic hepatitis B, *ACLF* acute-on-chronic liver failure, *M* male, *F* female, *AL* alanine aminotransferase, *AST* aspartate aminotransferase.

We hypothesized that extracellular gp96 may be involved in CHB disease progression and the development of liver failure. We therefore performed functional analysis of extracellular gp96 on immune hyperactivation-induced liver injury.

### LPS/d-Galn or ConA challenge induces an increase in serum gp96 levels

**Figure 2:**
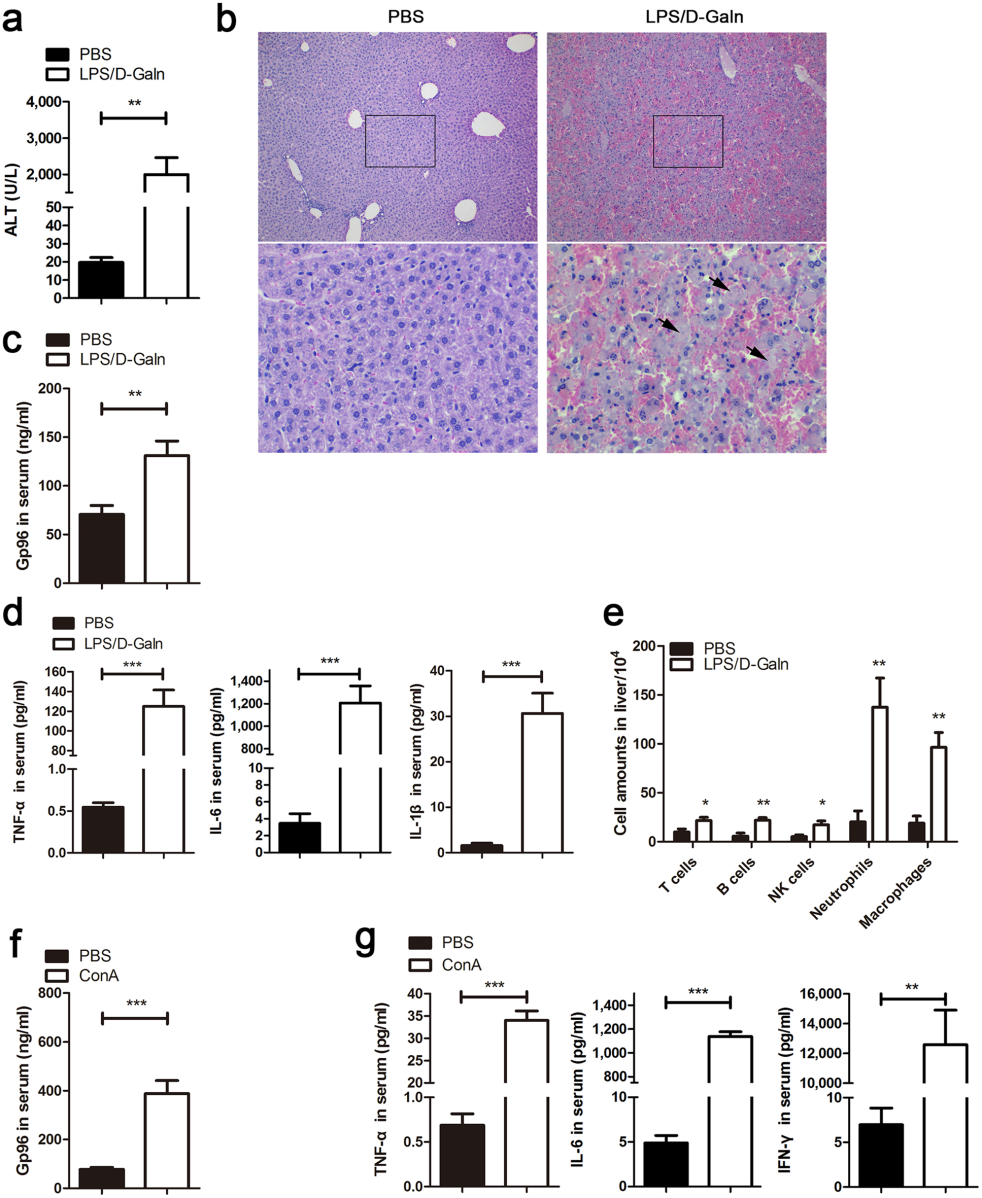
Serum gp96 levels are elevated in two mouse liver failure models. (**a**–**e**) Female C57 mice were challenged intraperitoneally (i.p.) with LPS (30 ng/g)/d-Galn (500 μg/g) or PBS as a control (n = 5/group). After 6 h, mice were sacrificed. Serum ALT levels (**a**) and liver histology (arrows indicate the necrotic areas) (**b**) were assessed. Serum gp96 (**c**) and pro-inflammatory cytokines including TNF-α, IL-6, and IL-1β (**d**) were quantified by ELISA. Intrahepatic immune cells were isolated, and then the total amounts of different cells were measured by flow cytometer (**e**). (**f**,**g**) Male BALB/c mice were challenged intravenously (i.v.) with ConA (15 μg/g) or PBS as a control (n = 5/group). After 8 h, mice were sacrificed. Serum gp96 (**f**) and pro-inflammatory cytokines including TNF-α, IL-6, and IFN-γ (**g**) levels were quantified by ELISA. Data are presented as mean ± SEM from two independent experiments.

We chose two classical mouse liver failure models induced by LPS plus d-Galn (LPS/d-Galn) or ConA to detect extracellular gp96 levels. Administration of LPS/d-Galn induced high levels of serum ALT (Fig. 2a), severe liver injury (Fig. 2b), high levels of inflammatory cytokines including TNF-α, IL-6, and IL-1β (Fig. 2d), and hepatic infiltration of immune cells including neutrophils, macrophages, T cells, B cells, and NK cells, (Fig. 2e). Although no obviously elevated expression of intracellular gp96 of hepatocytes was observed at 6 h after treatment with LPS/d-Galn by immunoblotting (Supplementary Fig. S2a), the serum gp96 level in mice was higher than that of control mice (Fig. 2c), indicating that extracellular gp96 may be released from necrotic hepatocytes. Similar results were observed in the ConA-induced mouse liver failure model. Extracellular serum gp96 levels were increased along with immune hyperactivation and liver injury (Fig. 2f,g and Supplementary Fig. S2b–d).

### Inhibition of gp96 results in the mitigation of LPS/d-Galn- or ConA-induced immune hyperactivation and liver injury

**Figure 3:**
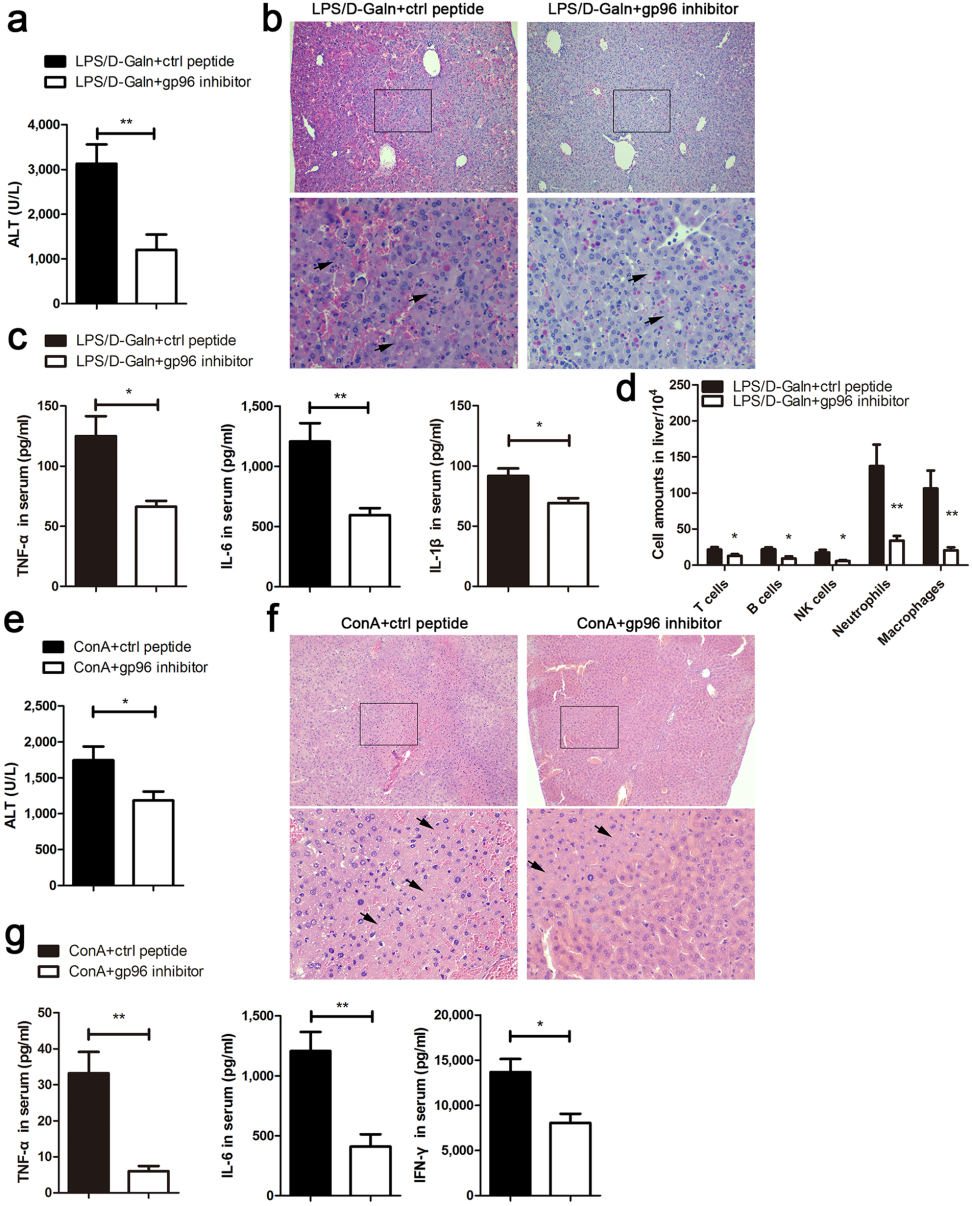
Inhibition of gp96 by the specific peptide mitigates immune hyperactivation and liver injury in mice induced by LPS/d-Galn or ConA. (**a**–**d**) Female C57 mice were challenged i.p. with LPS (30 ng/g)/d-Galn (500 μg/g). After 1 h, 100 μg of the gp96 inhibitor or control peptide was injected i.p. (n = 5/group). At 6 h after LPS/d-Galn treatment, mice were sacrificed. Serum ALT levels (**a**) and liver histology (arrows indicate the necrotic areas) (**b**) were assessed. Serum inflammatory cytokine (TNF-α, IL-6, and IL-1β) levels were measured by ELISA (**c**). Intrahepatic immune cells were isolated and analysed by flow cytometer (**d**). (**e**–**g**) Male BALB/c mice were challenged i.v. with ConA (15 μg/g). After 1 h, 100 μg of the gp96 inhibitor or control peptides was injected i.p. At 8 h after ConA treatment, mice were sacrificed. Serum ALT levels (**e**), liver histology (**f**), and serum inflammatory cytokine levels (**g**) were determined. Data are presented as mean ± SEM from three independent experiments.

As extracellular gp96 has been shown to display pro-inflammatory function in autoimmune diseases, we investigated whether inhibition of extracellular gp96 was able to limit immune hyperactivation and hepatic immunopathology in liver failure. A specific gp96 inhibitory peptide (amino acid 444–480 of gp96) from the middle domain of gp96 was used to target its N-terminal helix-loop-helix sequence, which specifically blocks gp96 intramolecular conformational changes by a trypsin-protection assay and multiple interaction analyses, as well as suppresses gp96 biological activities including its pro-inflammatory function^26,29–31^. A peptide derived from a α-helix adjacent to the N-terminal helix-loop-helix sequence (corresponding AA 61–100 of gp96) that has no obvious residue-residue contact within the gp96 molecule was used as a control. Treatment with the gp96 inhibitor led to a dramatic decrease (61.6%) in the ALT levels (Fig. 3a), apparent mitigation of the liver injury as determined by liver color darkness (Supplementary Fig. S3a) and histologic assessment of necrosis (Fig. 3b) in LPS/d-Galn-treated mice. In addition, compared to the control, inhibition of gp96 decreased serum TNF-α, IL-6, and IL-1β levels by 47.0%, 50.7%, and 24.7%, respectively (Fig. 3c).The number of intrahepatic neutrophils and macrophages, along with other immune cells was abruptly reduced by treatment with the gp96 inhibitor (Fig. 3d). Similarly, the protective effects were observed in ConA-treated mice by the gp96 inhibitor as determined by serum ALT levels (Fig. 3e), liver histological analysis (Fig. 3f), and the levels of key pro-inflammatory cytokines (Fig. 3g and Supplementary Fig. S3b). These data indicate that the reduced hepatic immunopathology was due to decreased hyperactivation by gp96 inhibition.

### Exogenous gp96 aggravates subdose LPS/d-Galn- or ConA-induced hepatic immunopathology

**Figure 4:**
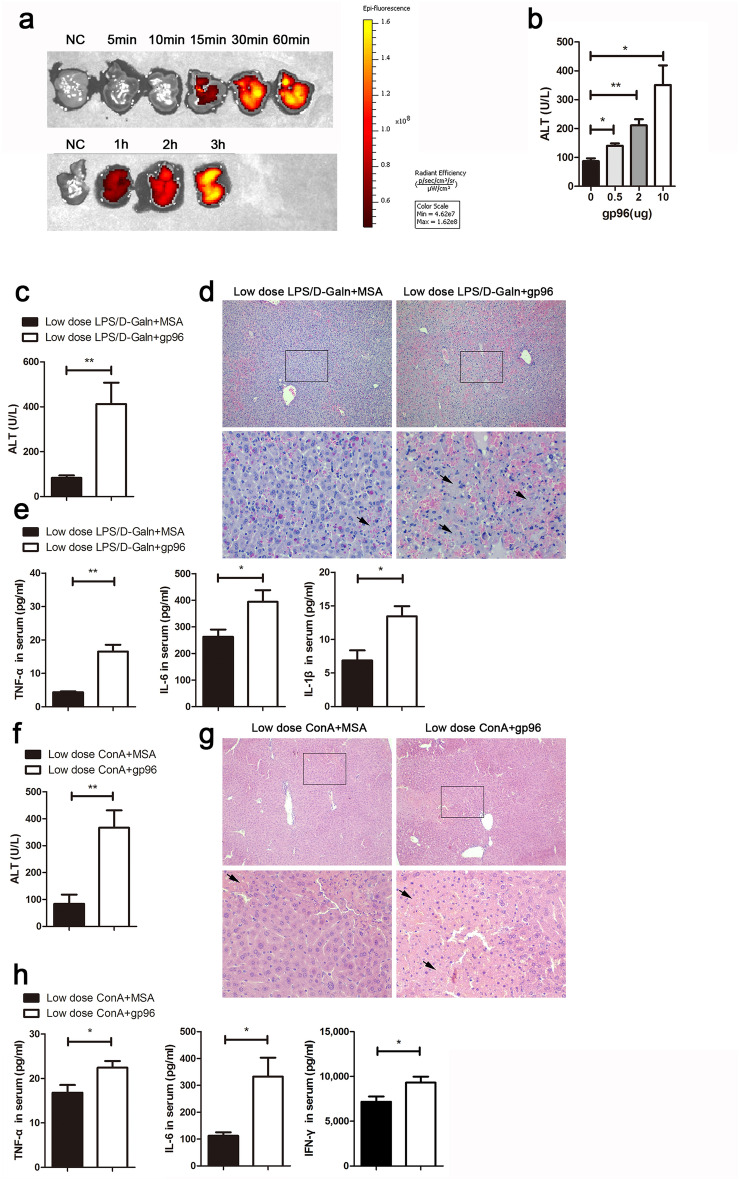
Exogenous gp96 promotes liver injury in mice after challenge with a low dose of LPS/d-Galn or ConA. (**a**) Female C57 mice were injected i.p. with 10 μg Cy5-labelled gp96 for different times as indicated before they were sacrificed. Photographs of livers were taken by IVIS spectrum. (**b**) Mice were injected i.p. with the indicated doses of gp96 respectively (n = 5/group) at 3 h after the challenge with a low dose of LPS (5 ng/g)/d-Galn (500 μg/g). The serum ALT levels was measured at 6 h after LPS/d-Galn treatment. (**c**–**e**) Female C57 mice were challenged i.p. with a low dose of LPS (5 ng/g)/d-Galn (500 μg/g). After 3 h, 2 μg gp96 or mouse serum albumin (MSA) were injected i.p. (n = 5/group). At 6 h after LPS/d-Galn treatment, mice were sacrificed. Serum ALT levels (**c**), liver histology (arrows indicate the necrotic areas) (**d**) was assessed. Serum inflammatory cytokine level were measured by ELISA (**e**). (**f**–**h**) Male BALB/c mice were challenged i.v. with a low dose of ConA (10 μg/g). After 5 h, 2 μg gp96 or MSA were injected i.p. (n = 5/group). At 8 h after ConA treatment, mice were sacrificed. Serum ALT levels (**f**), liver histology (arrows indicate the necrotic areas) (**g**) was assessed. Serum inflammatory cytokine level were measured by ELISA (**h**). Data are presented as mean ± SEM from two independent experiments.

We next tested if adding exogenous gp96 could aggravate the symptoms of liver failure. Cy5-labelled gp96 challenged intraperitoneally (i.p.) entered the mouse liver within 15 min and remained in the liver for at least 3 h (Fig. 4a). In addition, the administration of exogenous gp96 (0.5–10 μg/mouse) induced elevated ALT levels in a dose-dependent manner in mice treated with a low dose of LPS/d-Galn (Fig. 4b). A medium dose of gp96 (2 μg per mouse) that induced apparent ALT elevation was used for the following experiments.

As it is difficult to examine the effect of exogenous gp96 on normal dose of LPS/d-Galn- or ConA-induced hepatic immunopathology as in Fig. 2 (see Fig. 2 and Supplementary Fig. S4a,b), we used lower doses of reagents to induce less severe liver injury to determine if exogenous gp96 could aggravate hepatic immunopathology. As it can be seen, administration of exogenous gp96 promoted liver injury in both lower doses of LPS/d-Galn- and ConA-induced liver failure models, as evidenced by elevated serum ALT levels (Fig. 4c,f), obviously increased darkness of liver color (Supplementary Fig. S4c), and increased liver necrosis with the signature of loss of architecture (Fig. 4d,g). In addition, gp96-treated mice displayed elevated serum levels of key pro-inflammatory cytokines (Fig. 4e,h and Supplementary Fig. S4d).

### Gp96 mediates immune hyperactivation and liver damage in a Kupffer cell-dependent manner

**Figure 5:**
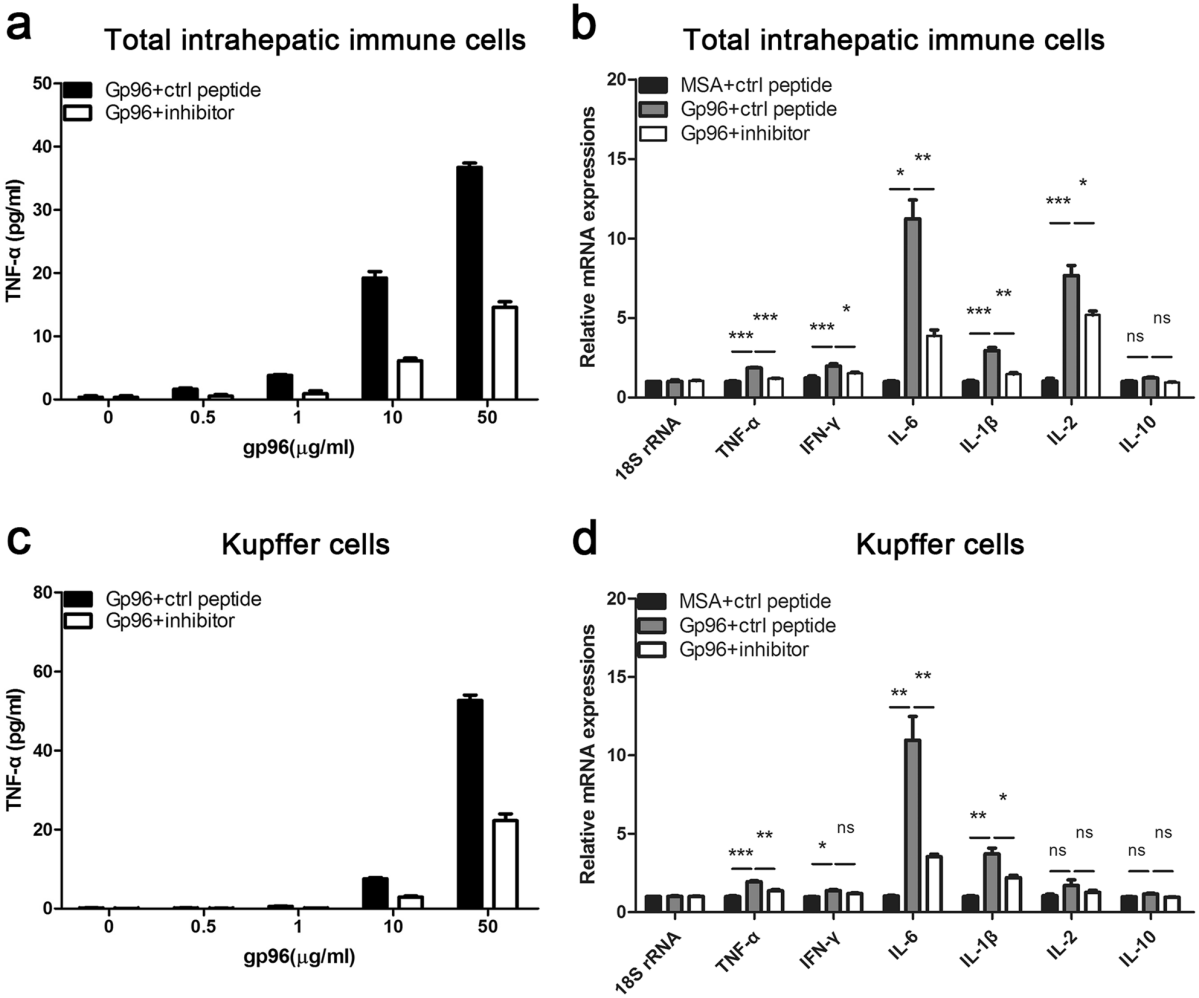
Gp96 induces expression of inflammatory cytokines of intrahepatic immune cells in vitro. (**a**,**c**) Total intrahepatic immune cells (**a**) or Kupffer cells (**c**) were isolated from the C57 mice and incubated with different doses of gp96 with or without the gp96 inhibitor. At 24 h after treatment, secreted TNF-α in the culture supernatant was detected by ELISA. (**b**,**d**) Total intrahepatic immune cells or Kupffer cells were treated with 10 μg/ml gp96 with or without the gp96 inhibitor(2 μg/ml) for 12 h. Relative mRNA expression of multiple inflammatory cytokines as indicated of total intrahepatic immune cells (**b**) or Kupffer cells (**d**) was detected by quantitative real-time PCR. Data are presented as mean ± SEM from three independent experiments. Ns, not significant, *p < 0.05, **p < 0.01, and ***p < 0.001 compared to the control.

Since dramatically increased infiltration of hepatic immune cells and released gp96 from necrotic hepatocytes were observed in LPS/d-Galn- and ConA-treated mice, we assumed that intrahepatic immune cells played important roles in immune hyperactivation. As shown in Fig. 5a, gp96 stimulated TNF-α secretion of total intrahepatic immune cells in a dose-dependent manner in vitro, which could be largely blocked by the gp96 inhibitor. The expression of key inflammatory cytokines including TNF-α, IFN-γ, IL-6, IL-1β, and IL-2 was considerably increased under gp96 stimulation (Fig. 5b). Similar stimulatory effects by gp96 were observed in Kupffer cells, one of the major hepatic pro-inflammatory immune cells during liver failure (Fig. 5c,d). Importantly, the stimulatory effects by gp96 could be largely blocked by the gp96 specific inhibitor. These results suggest extracellular gp96 may act as a pro-inflammatory mediator in hepatic hyperactivation.

**Figure 6:**
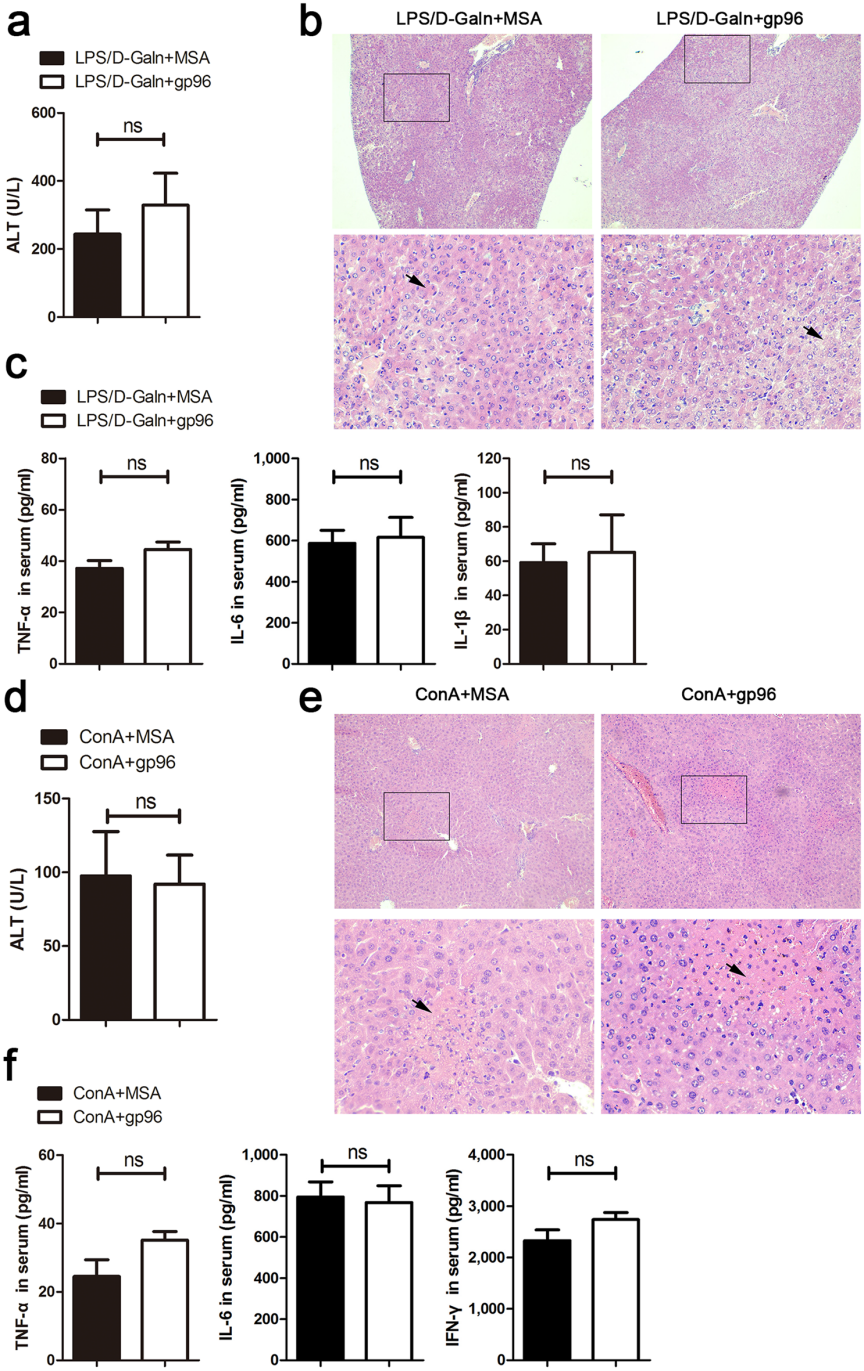
Exogenous gp96 loses its promotion effect on LPS/d-Galn- or ConA-induced liver injury in Kupffer cells-ablated mice. Mice were injected i.v. with 200 μl clodronate liposome for 48 h. (**a**–**c**) Female C57 mice were challenged i.p. with LPS (10 ng/g)/d-Galn (500 μg/g). After 3 h, 2 μg gp96 or MSA were injected i.p. (n = 5/group). At 6 h after LPS/d-Galn treatment, mice were sacrificed. Serum ALT levels (**a**), liver histology (arrows indicate the necrotic areas) (**b**), and serum inflammatory cytokine levels (**c**) were determined. (**d**–**f**) Male BALB/c mice were challenged i.v. with ConA (25 μg/g). After 5 h, 2 μg gp96 or MSA were injected i.p. (n = 5/group). At 8 h after ConA treatment, mice were sacrificed. Serum ALT levels (**d**), liver histology (**e**), and serum inflammatory cytokine levels (**f**) were assessed. Data are presented as mean ± SEM from two independent experiments.

As hepatic infiltration of macrophages was ameliorated by the gp96 inhibitor, and expression of pro-inflammatory cytokines by Kupffer cells was greatly enhanced under gp96 treatment (Figs. 3d, 5c,d), the potential role of Kupffer cells in gp96-mediated hepatic hyperactivation was further determined. We ablated Kupffer cells by treating mice with clodronate liposome. As shown in Supplementary Fig. S5a, Kupffer cells were largely depleted upon clodronate liposome treatment. Clodronate treatment greatly attenuated the increase of ALT and TNF-α by ConA or a relatively lower dose of LPS (10 ng/g) (see Supplementary Fig. S5b,c). However, no such effect was observed in mice treated with a higher dose of LPS (30 ng/g). Depletion of Kupffer cells in LPS/d-Galn-treated mice almost totally eliminated the impact of ALT elevation and aggravation of liver immunopathology by exogenous gp96 (Fig. 6a,b). In addition, no significant increase of production of pro-inflammatory cytokines was observed in gp96 treated mice compared to control mice (Fig. 6c). Similar results were observed in ConA-induced liver failure mouse model (Fig. 6d–f and Supplementary Fig. S5d). As it can be seen in Supplementary Figs. S3b and S5d, the TNF-α induced by ConA is much higher under treatment with gp96 inhibitor than with clodronate.

## Discussion

**Figure 7:**
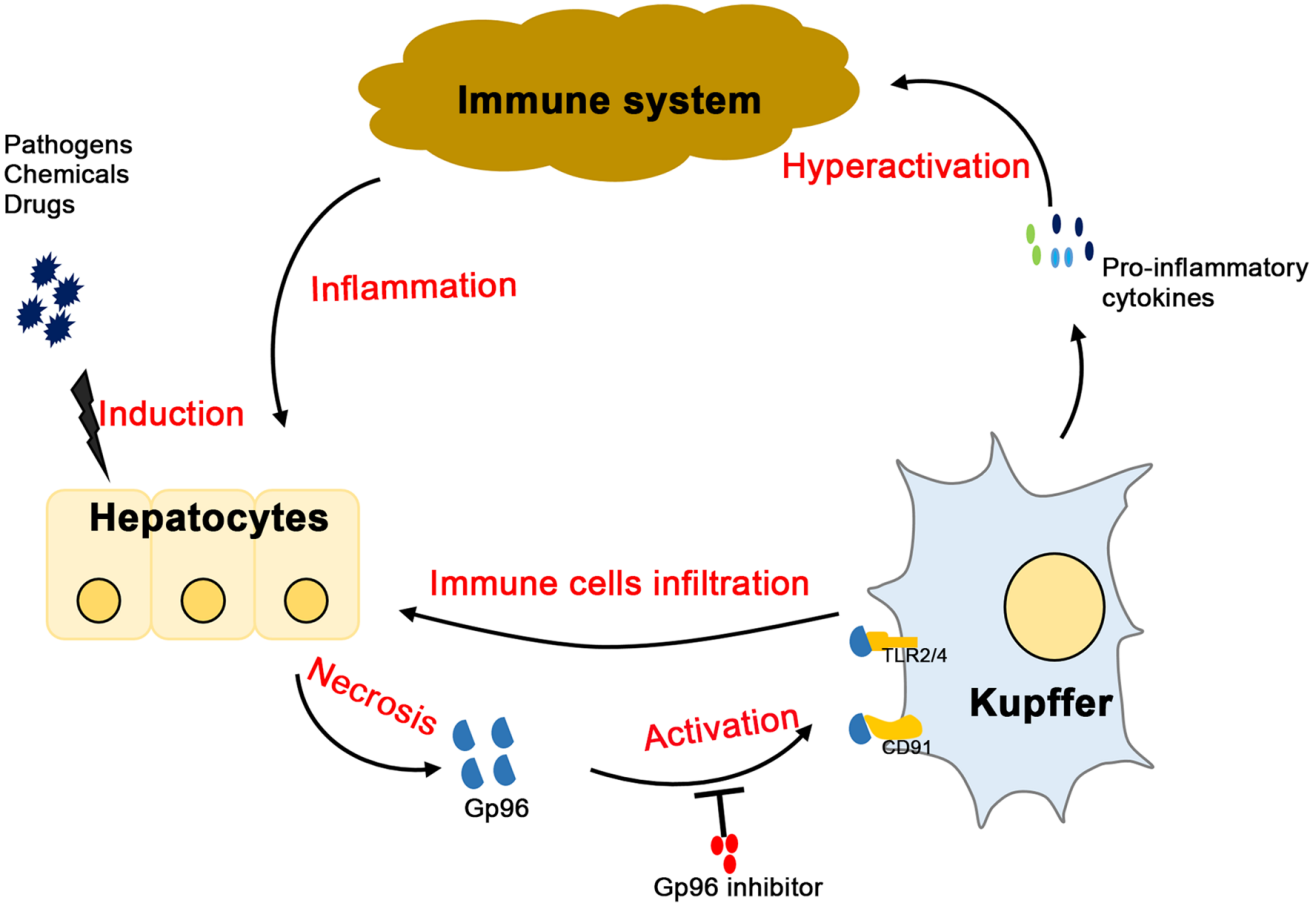
Schematic illustration of how extracellular gp96 promotes immune hyperactivation and liver damage during the development of liver failure. Multiple stimuli, including different pathogens, chemicals, or drugs, induces liver damage and necrosis of hepatocytes, which results in the release of extracellular gp96. Extracellular gp96 may recognize and bind to its receptors (e.g. CD91 or TLR2/4, etc.) to stimulate the activation of Kupffer cells, which secrete large amounts of pro-inflammatory cytokines, resulting in the infiltration of immune cells into the liver and hepatic hyperactivation. This may in turn lead to further damage.

Although emerging evidence demonstrates that DAMPs released after the massive necrosis of hepatocytes contribute to the development of liver failure^11,32,33^, the potential role of extracellular gp96 in immune-mediated liver damage during liver failure has not been determined. Here, we first found that serum gp96 levels in patients with CHB and ACLF were elevated, which correlated with clinically relevant parameters in patients with ACLF, including ALT levels and the key pro-inflammatory cytokines TNF-α and IL-6. Furthermore, increased gp96 in serum was also observed in mouse liver failure models. Next, our data demonstrated that exogenous gp96 aggravated liver injury and inflammation, whereas targeting extracellular gp96 with its specific inhibitor pronouncedly attenuated the symptoms of LPS/d-Galn- or ConA-induced hepatitis and liver failure. In addition, the effect of gp96 on the hepatic hyperactivation and liver damage in mice was mainly associated with activation of Kupffer cells. As shown in Fig. 7, according to this model, extracellular gp96 released during hepatic necrosis may act as a DAMP to activate Kupffer cells and contribute to further immune-mediated liver damage in the development of liver failure.

Inflammation occurs in response to a wide range of stimuli that causes liver injury, including the intoxication of xenobiotic (e.g., by para-acetaminophenol, APAP), cholesterol, alcohol, or hepatic ischemia–reperfusion injury (I/R). In inflammatory liver diseases, DAMPs released from dying cells play key roles in the activation of diverse immune cells by interacting with pattern recognition receptors, resulting in the production of inflammatory cytokines and hepatic relocalization of immune cells to the site of injury, which triggers further immune-induced liver damage^9^. The ability of extracellular gp96 as a DAMP to drive inflammation has been proven by a number of studies. It was previously found that gp96 could induce macrophages to secrete inflammatory cytokines^20^. Recently, gp96 was found to be able to activate the inflammasome-signaling platform in antigen presenting cells (APCs) even without additional stimuli^22^. In this study, the pro-inflammatory potential of gp96 was explored in liver failure models. Both co-incubation of gp96 with intrahepatic immune cells in vitro and injection of exogenous gp96 into mice promoted the production of pro-inflammatory cytokines, including TNF-α, IL-6, IL-1β, and IFN-γ. Importantly, targeting extracellular gp96 with its inhibitory peptide successfully mitigated the liver injury in ConA- and LPS/d-Galn-induced mouse liver failure models. Given that circulating gp96 exits predominantly in patients with CHB and ACLF but not in healthy individuals, we believe that inhibition or depletion of circulating and intrahepatic extracellular gp96 may provide an effective strategy for the treatment of liver hyperactivation and liver failure.

Liver failure is recognized as a complex syndrome with diverse pathogeneses and multiple complications. In the development of liver failure, the altered hepatic inflammatory status and systematic immune dysfunction, featured by apparent infiltration of multiple inflammatory cells (e.g., neutrophils and macrophages) in the liver and an abrupt increase of serum pro-inflammatory cytokines (e.g., TNF-α and IL-6), ultimately contribute to liver necrosis and liver failure^34–37^. Kupffer cells are liver resident macrophages and can promote the cellular infiltration of pro­inflammatory monocytes in the first place when an acute liver injury occurs^38^. The activated Kupffer cells are able to secrete a series of cytokines, chemokines, and complements^39^. Depletion of Kupffer cells has different impact on ConA- and LPS/d-Galn-induced liver failure model. Depletion of Kupffer cells greatly attenuated ConA-induced hepatitis. Clodronate liposome treatment attenuated the increase of ALT and TNF-α in mice challenged by a relatively lower dose of LPS (10 ng/g) but not in mice with a higher dose of LPS (30 ng/g). Similar trend was observed in a previous study^40^. We hypothesize that under lower dose LPS treatment Kupffer cells may also contribute to the increase of TNF-α in mice. This deserves further investigation. Along with the sensitivity of Kupffer cells to gp96 in vitro (Fig. 5b,c), we identified a hepatocyte necrosis-extracellular gp96-Kupffer cells-TNF-α/IL-6/IL-1β-regulatory loop in immune-mediated liver injury.

Extracellular gp96 in CHB and ACLF may be mainly released from immune-mediated necrotic cell death in the liver, but we cannot totally exclude the possibility that it might be released from intact cells^41,42^. In addition, currently it is difficult to totally mechanically distinguish the exact roles of gp96 and other potential DAMPs (e.g. other HSPs) released from necrotic hepatocytes in induction of the necroinflammation in liver failure, although inhibition of extracellular gp96 with its specific inhibitor was shown to largely attenuate immune hyperactivation and symptoms of liver failure in mice models. Of note, relatively higher serum gp96 levels in CHB were observed than those in ACLF (Fig. 1b,e), and exogenous gp96 promoted liver injury only in lower doses (Fig. 4) but not in normal doses (see Supplementary Fig. S4a,b) of LPS/d-Galn or ConA challenged mice models. We therefore speculate that reciprocal cross-talk between extracellular gp96, along with other DAMPs, other factors (e.g. drug, LPS, or alcohol) and multiple hepatic immune cells forms a positive feedback network in regulating hepatic hyperactivation and injury during liver failure. This deserves further investigation.

In conclusion, our findings highlight a novel role for extracellular gp96 after hepatocytes necrosis as a potent mediator of inflammatory responses, resulting in the aggravation of liver injury and possibly liver failure, in which the activation of Kupffer cells is mainly involved in this process. These findings underline the importance of extracellular gp96 as a potential therapeutic target in immune-induced liver damage, and blocking gp96 activity may be an effective approach against liver failure.

## Methods

### Animals

Female C57BL/6J mice aged 6 to 8 weeks old and male BABL/c mice aged 6 to 8 weeks old were purchased from SPF Biotechnology Company (Beijing, China). Mice were housed under controlled conditions of temperature (24 °C) with a light/dark cycle of 12/12 h, and with chow and water ad libitum. The experimental protocols used in animal studies were approved by the Institute of Microbiology, University of Chinese Academy of Sciences Ethics Committee. All methods were performed in accordance with the relevant guidelines of the University of Chinese Academy of Sciences.

### Model of liver failure

Acute liver injury was induced in female C57BL/6 mice by intraperitoneal (i.p.) injection of d-Galn (500 μg/g, Carl Roth, Karlsruhe, Germany) and LPS (30, 5 or 10 ng/g, Sigma-Aldrich, Hamburg, Germany) for 6 h or in male BABL/c mice by intravenous (i.v.) injection of concanavalin A (ConA) (15, 10 or 25 μg/g, Sigma-Aldrich, Hamburg, Germany) for 8 h.

### Patients

We collected serum samples from healthy volunteers (n = 8) and patients (n = 51) at The 5th Medical Centre, Chinese PLA General Hospital (Beijing, China). The patients included 35 individuals with acute-on-chronic liver failure (ACLF) and 16 with chronic hepatitis B (CHB). The standards for diagnosis of CHB and ACLF complied with the diagnostic criteria of the 2012 Diagnostic and Treatment Guidelines for Liver Failure issued by the Chinese Society of Infectious Diseases and Chinese Society of Hepatology, Chinese Medical Association. All serum samples were immediately snap frozen and stored at − 80 °C until analysis. All patients and healthy volunteers were provided with informed consent for biological research purposes. All protocols were approved by the Ethics Committee of The 5th Medical Centre, Chinese PLA General Hospital. All research activities were performed in accordance with the guidelines of the Declaration of Helsinki.

### Serological analysis

Serum gp96 levels were measured by enzyme-linked immunosorbent assay (ELISA). Serum alanine aminotransferase (ALT) activity was detected using a kinetic test (Rongsheng Biotech, Shanghai, China). Serum cytokines were quantified using ELISA kits (Invitrogen, CA, USA).

### Quantification of serum gp96

The serum gp96 was measured by ELISA. The capture antibody was purchased from Santa Cruz Biotechnology. The detection antibody for human gp96 was purchased from Aviva System Biology, and mouse gp96 was from Enzo Life Science. The horseradish peroxidase (HRP)-conjugated secondary antibody goat anti-rabbit IgG was from ZSGQ Biotechnology. The TMB was purchased from Invitrogen.

### Staining of immune cells

Cells were stained with the following fluorochrome-conjugated antibodies for 30 min at 4 °C in FACS staining buffer. The antibodies included anti-CD49b and anti-NK1.1 for NK cells, anti-CD3 for T cells, anti-CD19 for B cells, anti-CD11b and anti-Gr-1 for neutrophils, and anti-CD11b and anti-F4/80 for macrophages. All antibodies were from Invitrogen.

### Gp96-related reagents

Purification of gp96 protein from mouse liver and removal of endotoxin were performed as previously described^43,44^. The gp96 inhibitory peptide (LNVSRETLQQHKLLKVIRKKLVRKTLDMIKKIADDKY) and control peptide (LNASQIRELREKSEKFAFQAEVNRMMKLIINSLYKNKEIF) were chemically synthesized by Jier Biological Company (Shanghai, China). The purity (> 95%) and molecular weight of the peptides were determined by high-performance liquid chromatography and mass spectrometry.

### Isolation of immune cells

Primary mouse splenocytes were isolated as described previously^43^. Intrahepatic immune cells were isolated as previously described and subjected to flow cytometry and qRT-PCR^45^. Kupffer cells were isolated as described previously^46^.

### RNA extraction and quantitative real-time PCR (qRT-CPR)

Total RNA from immune cells co-incubated with gp96 was extracted using Trizol Reagent (Roche Diagnostics, Mannheim, Germany). The mRNA levels of cytokines were analysed by qRT-PCR using SYBR Premix Ex Taq (Takara, Dalian, China). Target gene expression was normalized to 18S rRNA (as an internal standard) levels. PCR primer sequences are shown in Supplementary Table S1.

### Histology and immunohistochemistry (IHC)

Paraffin-embedded liver sections of 4 µm thickness were prepared from liver tissue, fixed in 10% (v/v) neutral-buffered formalin. Sections were stained with haematoxylin & eosin (H&E) for histological examination and gp96 antibody to detect the expression of gp96 in the liver.

### In vivo imaging study (IVIS)

Mice were injected i.p. with 10 μg Cy5-gp96. At different time points, mice were sacrificed to remove the livers, and images were recorded using an IVIS Imaging System (Xenogen, USA). The bioluminescence images were quantified with Living Image software (Xenogen, USA).

### Kupffer cells depletion

To deplete Kupffer cells, mice were given clodronate liposomes (YEASEN, Shanghai, China) injections (200 μl per mouse) intravenously 48 h prior to the induction of liver failure.

### Statistical analysis

The statistical significance of differences was determined by two tailed Student's *t*-test. Differences with a p-value < 0.05 were considered to be statistically significant. Pearson's correlation coefficients were used to evaluate correlations between two variables.

## Supplementary information

Supplementary information.

## Data Availability

All data generated or analysed during this study are included in this published article (and its Supplementary Information Files). The described materials are freely available for scientific purposes.
