# Supplementary material for: Extracellular gp96 is a crucial mediator for driving immune hyperactivation and liver damage
Source: Sci Rep. 2020 Jul 28;10:12596. doi: 10.1038/s41598-020-69517-7 (PMC7387550; doi:10.1038/s41598-020-69517-7)
Supplement: Supplementary file 1 — Supplementary information. [file 41598_2020_69517_MOESM1_ESM.pdf]

Extracellular gp96 is a crucial mediator for driving immune hyperactivation and liver damage

Zeliang Guan[1, 2], Yun Ding[3], Yongai Liu[1, 2], Yu Zhang[4], Jingmin Zhao[4], Changfei Li[1, 2]*, Zihai Li[5], and Songdong Meng[1, 2]*

[1]Key Laboratory of Pathogenic Microbiology and Immunology, Institute of Microbiology, Center for Biosafety Mega-Science, Chinese Academy of Sciences (CAS), Beijing, China;
[2]University of Chinese Academy of Sciences, Beijing, China;
[3]Heilongjiang Bayi Agricultural University, Heilongjiang, China;
[4]302 Military Hospital of China, Beijing, China;
[5] Pelotonia Institute for Immuno-Oncology, The Ohio State University, Columbus, Ohio, USA

*Corresponding Authors. mengsd@im.ac.cn; lichangfei2006@163.com

Supplementary Table. S1： Primers sequences for qRT-PCR

18S rRNA-F	GTTCCGACCATAAACGATGCC
18S rRNA-R	TGGTGGTGCCCTTCCGTCAAT
TNFα-F	AAATTCGAGTGACAAGCCTGTAGCC
TNFα -R	AGTTGGTTGTCTTTGAGATCCATGC
IFN γ-F	CTCATGGCTGTTTCTGGCTGTTACT
IFN γ -R	GCCAGTTCCTCCAGATATCCAAGA
IL6 F	AGTTGCCTTCTTGGGACTGA
IL6 R	TCCACGATTTCCCAGAGAAC
IL1β -F	AGCTGAAAGCTCTCCACCTCAATG
IL1 β -R	CACAGGTATTTTGTCGTTGCTTGGT
IL2-F	CCTGAGCAGGATGGAGAATTACA
IL2-R	TCCAGAACATGCCGCAGAG
IL10-F	CAGTACAGCCGGGAAGACAAT
IL10-R	TGGCAACCCAAGTAACCCTTA

Supplementary Table. S2: Antibodies

Antigen	Company	Catalog	Application
Gp96	Santa Cruz	sc-32249	WB,IHC
Gp96	Enzo life	ADI-SPA-851-D	ELISA
Gp96	Aviva system	ARP40463_P050	ELISA
GAPDH	Santa Cruz	sc-32233	WB
CD3	eBioscience	11-0031-63	FC
CD4	eBioscience	12-0041-81	FC
CD8	eBioscience	45-0081-80	FC
CD11b	eBioscience	12-0112-81	FC
CD19	eBioscience	12-0193-81	FC
F4/80	eBioscience	45-4801-80	FC
CD49b	eBioscience	11-5971-81	FC
Gr-1	eBioscience	17-5931-81	FC
NK 1.1	eBioscience	17-5941-81	FC

Supplementary Figure S1

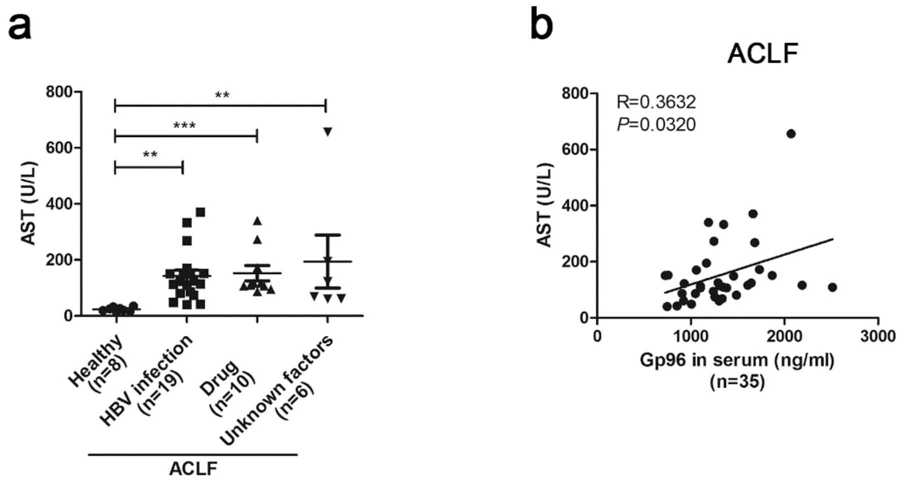

Supplementary Figure S1. Correlation analysis between serum AST and gp96 levels in patients with ACLF. (**a**) Serum aspartate aminotransferase (AST) levels of patients with ACLF. (**b**) Correlation analysis between serum AST and gp96 levels in patients with ACLF. Pearson's correlation coefficient (R) and *P-value* were analyzed. Data are presented as mean ± SEM. ** p<0.01, and *** p<0.001 compared to the control.

Supplementary Figure S2

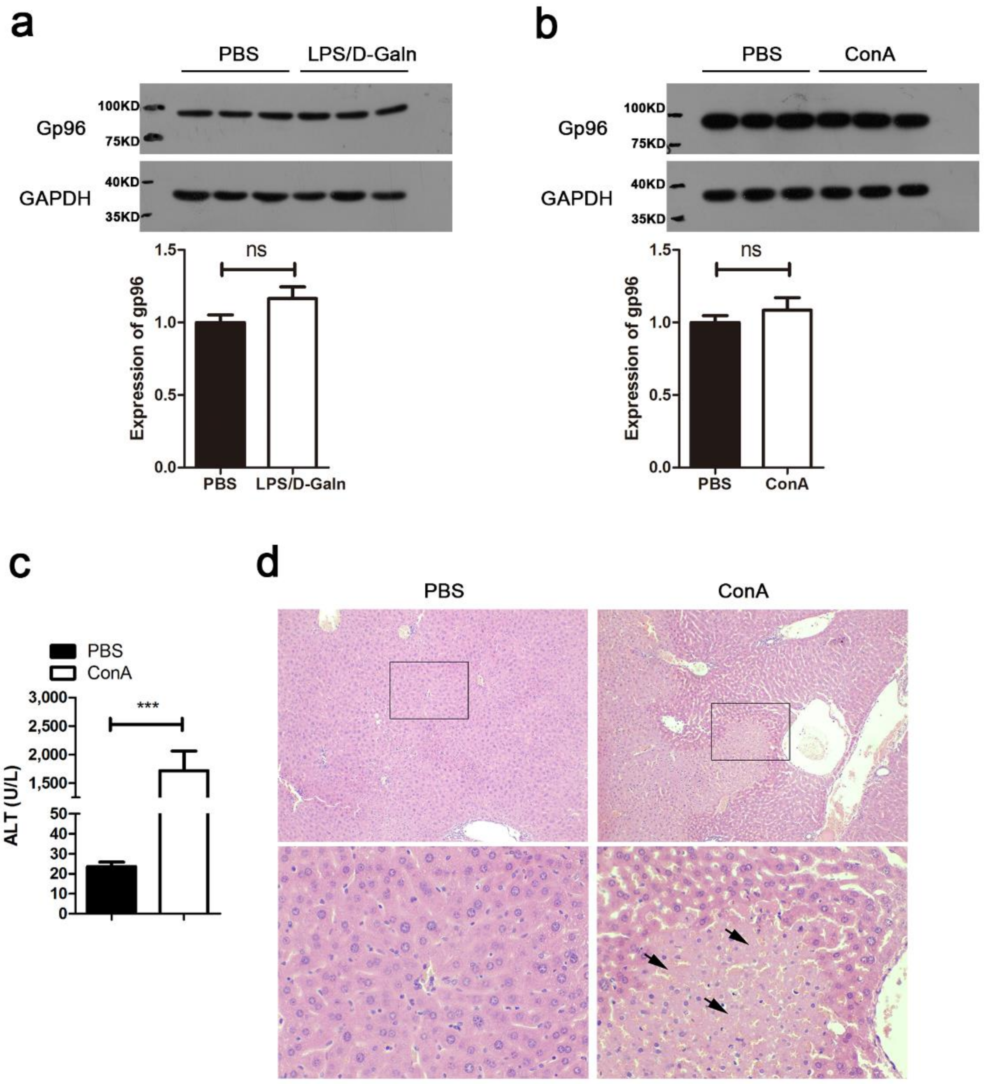

Supplementary Figure S2. Challenge with LPS/D-Galn or ConA has no obvious effect on intracellular gp96 expression. (**a**) Six hours after the challenge i.p. with LPS (30 ng/g)/D-Galn (500 μg/g), livers were isolated to measure the expression of intracellular gp96 by immunoblotting. Full-length blots are presented in Supplementary Figure S6. (**b**-**d**) Eight hours after the challenge i.v. with ConA (15 μg/g), mice were sacrificed. Expression of intracellular gp96 were measured by immunoblotting (**b**). Full-length blots are presented in Supplementary information 2.

Serum ALT levels (**c**) and liver histology (arrows indicate the necrotic areas) (**d**) were assessed.

Supplementary Figure S3

a

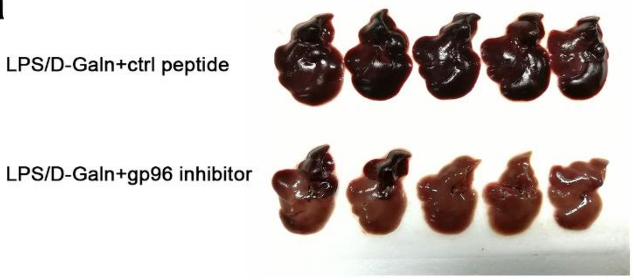

b

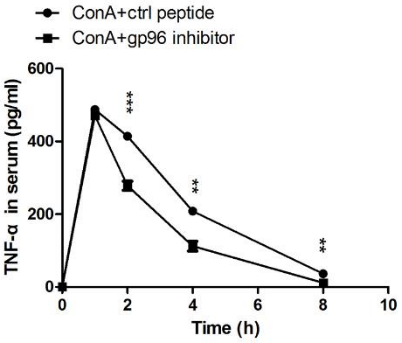

Supplementary Figure S3. Inhibition of gp96 mitigated liver injury induced by LPS/D-Galn or ConA. (**a**) Female C57 mice were challenged i.p. with LPS (30 ng/g)/D-Galn (500 μg/g). After 1 h, the mice were randomly divided into two groups, and 100 μg of gp96 inhibitor or control peptide was injected i.p (n=5/group). At 6 h after LPS/D-Galn treatment, mice were sacrificed. Photographs of livers were taken. (**b**) Male BALB/c mice were challenged i.v. with ConA (15 μg/g). After 1 h, 100 μg of the gp96 inhibitor or control peptide was injected i.p (n=5/group). Serum TNF-α levels at 1 h, 2 h, 4 h, and 8 h were determined.

Supplementary Figure S4

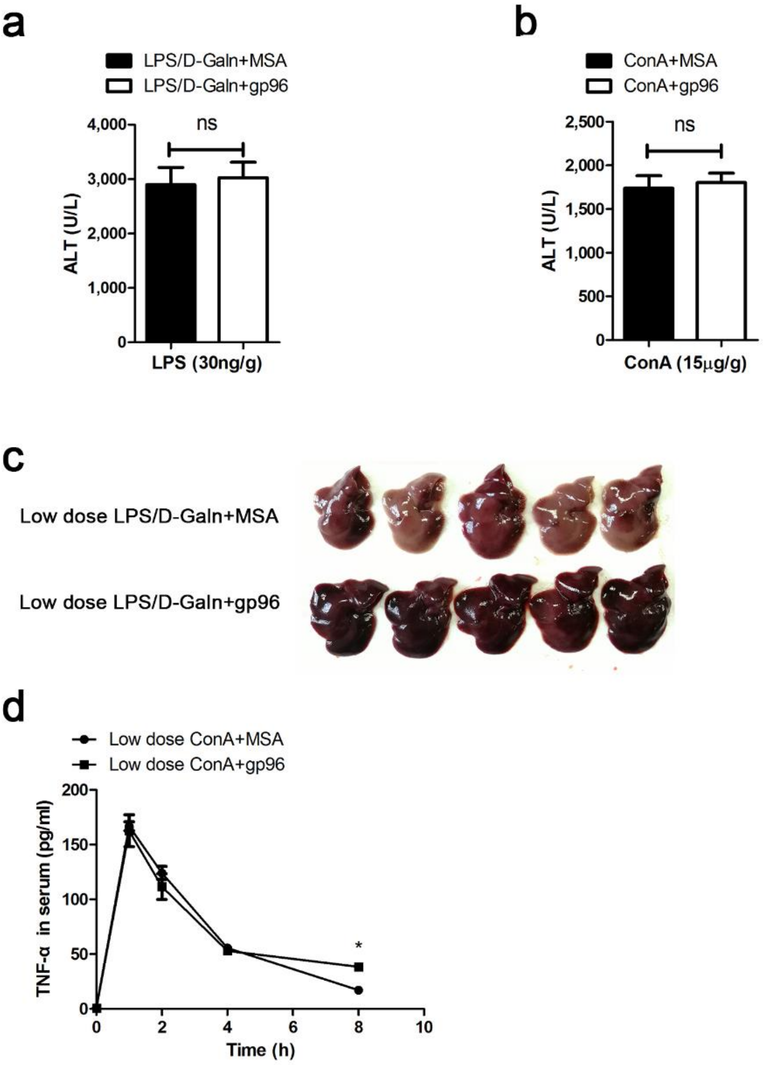

Supplementary Figure S4. Exogenous gp96 aggravates liver injury after challenge with a low dose of LPS/D-Galn or ConA. (**a**, **c**) Female C57 mice were challenged i.p. with LPS (30 ng/g (**a**) or 5 ng/g (**c**))/D-Galn (500 μg/g). After 3 h, 2 μg recombinant gp96 or mouse serum albumin (MSA) were injected i.p (n=5/group). At 6 h after LPS/D-Galn treatment, mice were sacrificed. (**a**) Photographs of livers were taken. (**c**) Serum ALT was measured. (**b**, **d**) Male BALB/c mice were challenged i.v. with ConA (15 μg/g (**b**) or 10 μg/g (**d**)). After 5 h, 2 μg gp96 or MSA were injected i.p.

(n=5/group). (**b**) Serum ALT was measured. (**d**)Serum TNF-α levels at 1 h, 2 h, 4 h, and 8 h were determined.

Supplementary Figure S5

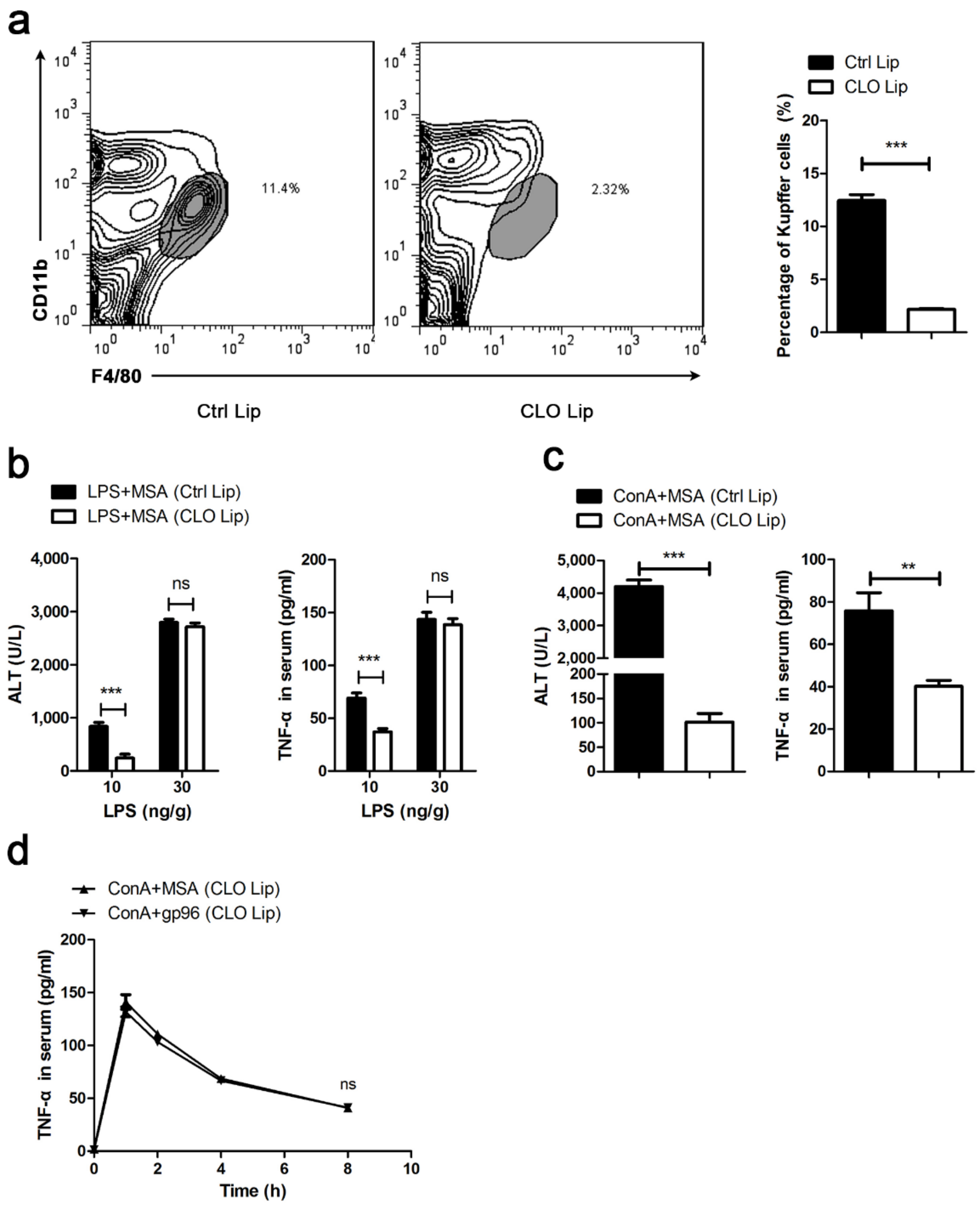

Supplementary Figure S5. Exogenous gp96 loses its promotion effect on liver injury after challenge with LPS/D-Galn in Kupffer cells-ablated mice. (**a**) Female

C57 mice were injected intravenously with 200μl clodronate liposome (CLO Lip) or control liposome (Ctrl Lip) for 48h. Then total intrahepatic immune cells were isolated and stained with CD11b and F4/80 antibody. (**b**) Female C57 mice with or without pretreatment of clodronate liposome for 48 h were challenged i.p. with LPS (10 or 30 ng/g)/D-Galn (500 μg/g). After 3 h, 2 μg mouse serum albumin (MSA) were injected i.p. At 6 h, mice were sacrificed. Serum ALT and TNF-α levels were determined. (**c**) Male BALB/c mice with or without pretreatment of 200μl clodronate liposome for 48 h were challenged i.v. with ConA (25 μg/g). After 5 h, 2 μg MSA were injected i.p. At 8 h, mice were sacrificed. Serum ALT and TNF-α levels were determined. (**d**) Male BALB/c mice with pretreatment of 200μl clodronate liposome for 48 h were challenged i.v. with ConA (25 μg/g). After 5 h, 2 μg gp96 or MSA were injected i.p. (n=5/group). Serum TNF-α levels at 1 h, 2 h, 4 h, and 8 h were determined.

Supplementary Figure S6

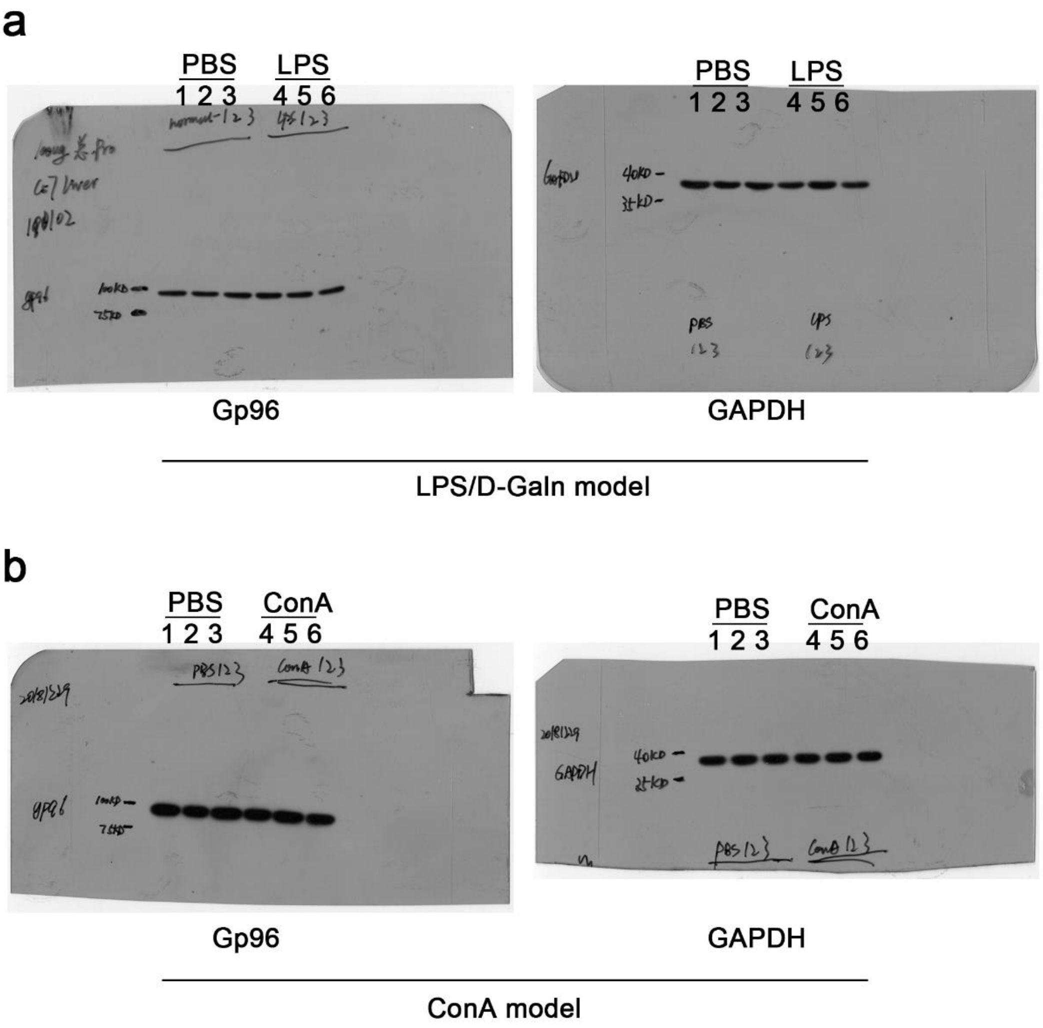

Supplementary Figure S6. Full length blots of Supplementary Figure S2a (**a**)and S2b (**b**) are presented.
